# Prothrombotic fibrin clot properties associated with NETs formation characterize acute pulmonary embolism patients with higher mortality risk

**DOI:** 10.1038/s41598-020-68375-7

**Published:** 2020-07-10

**Authors:** Michał Ząbczyk, Joanna Natorska, Agnieszka Janion-Sadowska, Agnieszka Metzgier-Gumiela, Mateusz Polak, Krzysztof Plens, Marianna Janion, Grzegorz Skonieczny, Katarzyna Mizia-Stec, Anetta Undas

**Affiliations:** 1 Institute of Cardiology, Jagiellonian University Medical College, 80 Pradnicka St.,, 31-202 Krakow, Poland; 2 John Paul II Hospital, Krakow, Poland; 3 The Faculty of Medicine and Health Sciences, The Jan Kochanowski University, Kielce, Poland; 4 Provincial Polyclinical Hospital, Torun, Poland; 5 First Department of Cardiology, Leszek Giec Upper-Silesian Medical Centre of the Silesian Medical University, Katowice, Poland; 6 KCRI, Krakow, Poland

**Keywords:** Medical research, Biomarkers, Embolism, Thromboembolism, Thrombosis

## Abstract

Venous thromboembolism is associated with formation of denser fibrin clots resistant to lysis. We investigated whether prothrombotic plasma clot properties are associated with the severity of acute pulmonary embolism (PE). We enrolled 126 normotensive acute PE patients (aged 58 ± 14 years) and 25 age- and sex-matched healthy controls. Plasma fibrin clot permeability (K_s_), clot lysis time (CLT), endogenous thrombin potential (ETP), plasminogen activator inhibitor-1 (PAI-1), and citrullinated histone H3 (citH3) were evaluated on admission. PE patients compared to controls had 370% higher citH3 levels, 41% higher ETP, 16.5% reduced K_s_, and 25.6% prolonged CLT. Patients with intermediate-high (n = 29) and intermediate-low (n = 77) PE mortality risk had reduced K_s_ and prolonged CLT, increased PAI-1 and ETP as compared to low-risk PE (n = 20) patients. Prolonged CLT was predicted by PAI-1 and citH3, while low K_s_ by C-reactive protein. During a 12-month follow-up 9 (7.1%) patients who had 24% higher ETP, 45% higher citH3 levels, and 18% prolonged CLT at baseline died. High ETP combined with elevated citH3 levels and prolonged CLT was associated with eightfold increased risk of PE-related death. Prothrombotic fibrin clot properties and enhanced neutrophil extracellular traps formation are associated with higher early mortality risk in acute PE patients, which suggests a prognostic role of these biomarkers.

## Introduction

Acute pulmonary embolism (PE), isolated and combined with deep-vein thrombosis (DVT), remains a major cause of mortality, morbidity, and hospitalization related to venous thromboembolism (VTE)^1^. PE occurs when a blood clot, mostly originating from proximal veins of the lower extremities, breaks free and flows to the pulmonary arteries blocking the blood flow^2^. PE is associated with 30-day all-cause mortality rate about 10% and three-month mortality up to 17%^3^. Clinical prediction scores have been developed to standardize diagnosis and assess prognosis in acute PE^3^. Intermediate-risk PE is associated with mortality ranging from 3 to 14%, while high-risk PE up to 24%^4^. The pathogenesis of PE is multifactorial involving episodes provoked by cancer, surgery, hospitalization, estrogen use, along with unprovoked events in > 50% of acute PE patients^3,4^. Recently, VTE has been linked to the release of neutrophil extracellular traps (NETs)^5^. Extracellular chromatin and citrullinated histones H3 (citH3) have been observed in deep-vein thrombi in mice^6^ and VTE patients^7^. NETs formation markers were significantly elevated in symptomatic VTE patients compared to healthy controls^5^, but NETs formation has not been explored in patients with acute PE so far.

Growing evidence indicates that a prothrombotic clot phenotype, characterized by the formation of compact fibrin clots relatively resistant to lysis, can be detected in patients with arterial and venous thromboembolic events^8,9^, including those following PE^10,11^. Faster fibrin degradation has been shown in vitro in patients following central versus peripheral PE^12^. It has been reported that prothrombotic clot properties may predispose to recurrent PE in patients after the first-ever PE episode^13^. Data about fibrin clot properties in patients with acute PE are limited. Martinez et al.^14^ have shown faster clot lysis time and earlier establishment of clot viscoelastic properties among 40 acute PE patients as compared to 35 patients with DVT alone. It is unknown whether fibrin clot properties assessed on admission in patients with acute PE can reflect a disease severity and may have a prognostic value. Therefore, we investigated plasma clot properties in acute PE and their association with the severity of PE.

## Material and methods

### Patients

A total of 187 consecutive adult white patients with acute symptomatic PE were recruited from December 2016 to March 2019, all emergency admissions, together with concurrently enrolled 25 age- and sex-matched healthy controls. The diagnosis of PE was based on the presence of typical symptoms and positive results of high resolution spiral computed tomography (CT). Based on the prespecified criteria, we excluded 61 subjects, due to known cancer (n = 12), pregnancy/postpartum (n = 11), high-risk PE with shock or hypotension (n = 6), ischemic stroke in the past 3 months (n = 5), myocardial infarction in the past 3 months (n = 2), end-stage kidney disease (n = 2), vitamin K antagonist use (n = 2), and 21 due to anti-Xa activity ≥ 0.2 U/mL (arbitrarily chosen as a cut-off value based on our previous study)^15^, because patients who received thromboprophylaxis with low-molecular-weight heparins within 12 h were eligible since the diagnosis of PE indicated either non-compliance or ineffective management.

We assessed the simplified PE severity index (sPESI)^3^. Low-risk PE was indicated by the sPESI of 0. Normotensive PE patients with sPESI of ≥ 1 represented an intermediate-risk group. Using the ESC approach, patients with both right ventricular (RV) dysfunction (by echocardiography or CT angiography) and elevated cardiac troponin T (TnT) represented an intermediate-high-risk category, while those with normal RV or TnT levels were classified as an intermediate-low-risk group. RV dysfunction was defined as previously described^16^. For definition of DVT, unprovoked VTE, heart failure (HF), and chronic obstructive pulmonary disease (COPD) see the Supplementary Material. All participants were contacted at least twice a year through clinic visits after 3 and 12 months. The primary composite endpoint was the occurrence of recurrent PE and/or DVT or death. The Jagiellonian University Ethical Committee approved the study, and participants provided informed written consent. All methods were carried out in accordance with relevant guidelines and regulations.

### Laboratory investigations

Before initiation of anticoagulant therapy on admission, blood samples were drawn from an antecubital vein with minimal stasis. Blood cell count, creatinine, prothrombin time (international normalized ratio, INR), activated partial thromboplastin time (aPTT), lipid profile, d-dimer, N-terminal B-type natriuretic propeptide (NT-proBNP), and high-sensitivity TnT were assayed by routine laboratory techniques in the hospital laboratory. Positive high-sensitivity TnT was defined as a value ≥ 14 pg/mL and positive NT-proBNP as a value ≥ 600 pg/mL^3^. A chromogenic assay was used to measure anti-Xa activity (BIOPHEN, Hyphen-Biomed, Neuville-Sur-Oise, France). Fibrinogen was determined using the Clauss method. C-reactive protein (CRP) was measured by immunoturbidimetry (Roche Diagnostics, Mannheim, Germany). Plasminogen activator inhibitor-1 (PAI-1) and citH3 levels were evaluated using chromogenic or ELISA tests (see Supplementary Material).

For fibrin clot analyses, blood samples (vol/vol, 9:1 of 3.2% trisodium citrate) were spun at 2500 g for 10 min and the supernatant was aliquoted and stored at − 80 °C^11,15^. All measurements were performed by technicians blinded to the origin of the samples^11,15^. Intra-assay and inter-assay coefficients of variation were 5–7%^13^. Fibrin clot permeation was determined using a pressure-driven system^11^. After K_s_ measurement, clots were analysed using scanning electron microscopy (SEM)^13^. To assess efficiency of clot lysis, clot lysis time (CLT) was measured using the assay of Pieters et al.^17^. Endogenous thrombin potential (ETP) was performed using calibrated automated thrombography (Thrombinoscope BV, Maastricht, the Netherlands). For details see the Supplementary Material.

### Statistical analysis

Variables are presented as numbers and percentages, mean ± standard deviation (SD) or median and interquartile range (IQR), as appropriate^15^. Normality was assessed by Shapiro–Wilk test^15^. Equality of variances was assessed using the Levene’s test. Differences between groups were compared using the Student’s or the Welch’s t-test depending on the equality of variances for normally distributed variables^15^. The Mann–Whitney U test was used for non-normally distributed variables^15^. Categorical variables were compared by the Pearson’s chi-squared test or Fisher’s exact test^15^. Multiple group comparisons were performed using analysis of variance (ANOVA) or Kruskal–Wallis test. Tukey–Kramer HSD test or Steel–Dwass method was used for the post-hoc comparisons. For multiple comparisons of categorical parameters the Benjamini–Hochberg procedure was used. Univariate and multivariate logistic regression models were performed to identify independent predictors of prolonged CLT or reduced K_s_ (see the Supplementary Material). The best cut-off value that maximizes sensitivity and specificity and differentiates PE patients who died from survivors was calculated by using the Receiver Operating Characteristics (ROC) curve. The study was powered to have a 90% chance of detecting a 10% difference in fibrin clot characteristics using a p-value of 0.05^13^. All statistical analyses were performed with JMP, Version 13.1.0 (SAS Institute INC., Cary, NC, USA).

## Results

Patient characteristics compared to age- and sex-matched healthy controls are presented in Table S1. Median time from the PE symptoms was 4 days (range 0–22 days). Provoked PE was diagnosed in 45 patients, including 6 (4.8%) women taking oral contraceptives or hormone replacement therapy. In 42 (33.3%) PE patients positive TnT was found, while 47 (37.3%) patients had positive NT-proBNP. RV dysfunction was observed in 46 patients (36.5%). Compared to controls, acute PE patients had 370% higher citH3 levels, 41% higher ETP and showed prothrombotic fibrin clot phenotype as reflected by 16.5% reduced K_s_ and 25.6% prolonged CLT (Table S1).

### Mortality risk prediction scores

**Figure 1:**
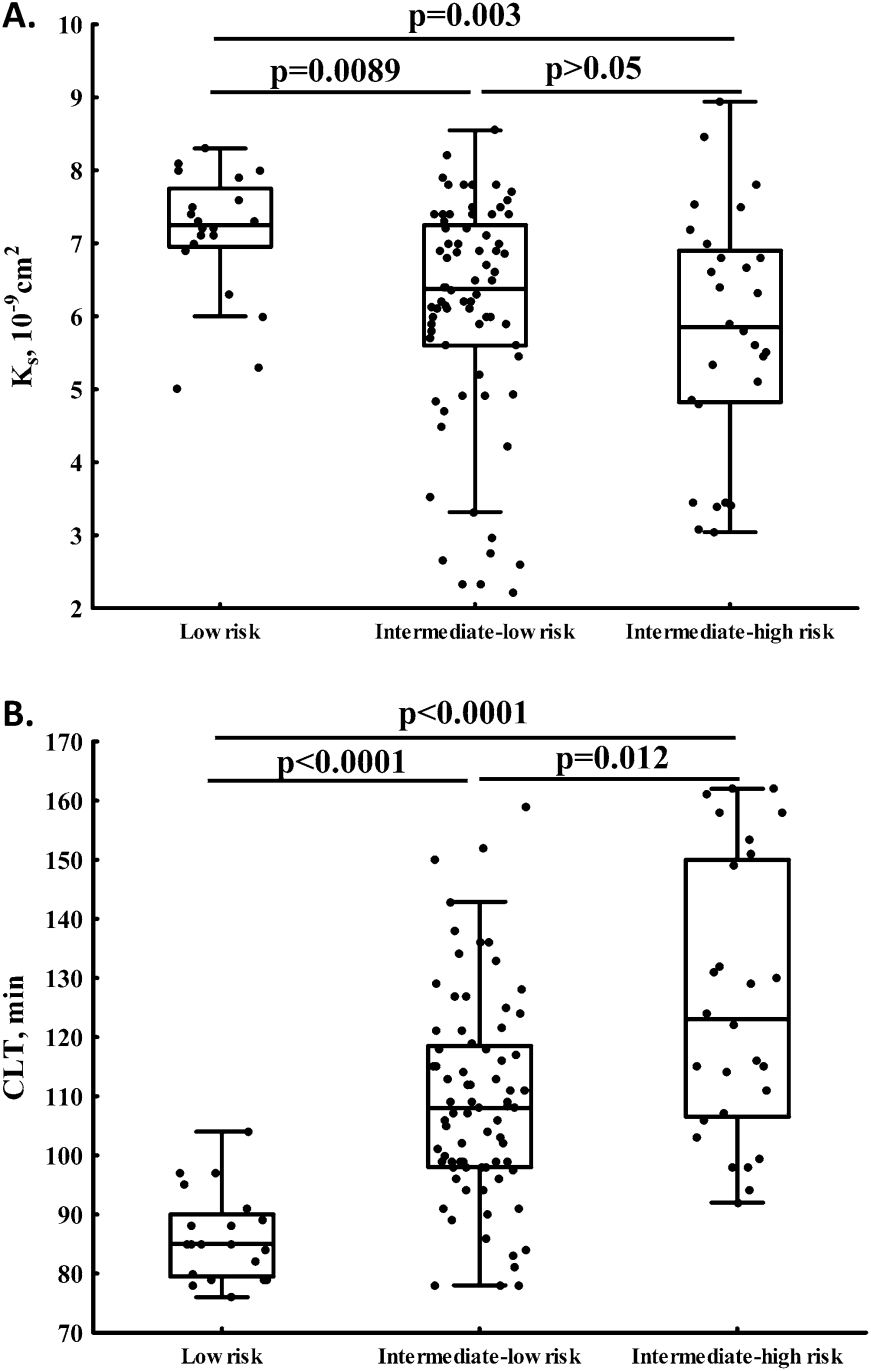
Fibrin clot permeability (K_s_, **A**) and clot lysis time (CLT, **B**) determined in acute PE patients with low, intermediate-low, and intermediate-high early mortality risk (both p < 0.05 for ANOVA).

There were 29 (23%) patients with intermediate-high risk PE, 77 (61.1%) with intermediate-low risk, and 20 (15.9%) subjects with low-risk PE (Table 1). PE patients at intermediate-high or -low vs. low mortality risk did not differ with regard to demographic and clinical variables, except higher incidence of HF and COPD, and lower prevalence of prior VTE in subjects with higher mortality risk (Table 1). Of laboratory investigations, NT-proBNP, PAI-1, and ETP increased with higher mortality risk score (Table 1). Intermediate-high risk PE was associated with 19.2% reduced K_s_ and 43.5% prolonged CLT and in patients with intermediate-low mortality risk K_s_ was 12.3% lower, while CLT was 27.1% longer as compared to those with low risk (Fig. 1A,B).

**Table 1:** Characteristics of patients with acute pulmonary embolism (PE) according to low, intermediate-low, and intermediate-high early mortality risk.

Variable	Early mortality risk		
	Low risk (n = 20)	Intermediate-low risk (n = 77)	Intermediate-high risk (n = 29)
Age, years	55 ± 12.7	55.6 ± 14.8	67.2 ± 10.7
Men, n (%)	13 (65)	40 (51.9)	13 (44.8)
Body-mass index, kg/m^2^	28.6 ± 5.2	27.9 ± 4.6	28.5 ± 6
Current smoking, n (%)	4 (20)	14 (18.2)	7 (24.1)
**Clinical characteristics, n (%)**			
Prior venous thromboembolism	3 (15)*	6 (7.8)*^†^	0*^#^
Coronary heart disease	7 (35)	33 (42.9)	10 (34.5)
Prior myocardial infarction	1 (5)	19 (24.7)	5 (17.2)
Prior stroke	1 (5)	6 (7.79)	3 (10.3)
Hypertension	9 (45)	38 (49.4)	21 (72.4)
Heart failure	0*	17 (22)*^†^	8 (27.6)*^#^
Diabetes mellitus	6 (30)	26 (33.8)	10 (34.5)
COPD	0*	8 (10.4)*^†^	4 (13.8)*^#^
**Medications, n (%)**			
Beta blockers	20 (100)*	50 (64.9)*^†^	21 (72.4)*^#^
ACEI	12 (60)	42 (54.5)	20 (69)
ARB	5 (25)*	11 (14.3)*	0*^‡#^
Calcium channel blockers	1 (5)	11 (14.3)	4 (13.8)
Aspirin	5 (25)	25 (32.5)	10 (34.5)
Statin	13 (65)	46 (59.7)	18 (62.1)
**Characteristics of acute PE**			
PE symptoms, days	3.5 [1.5–7]	4 [2–7]	3 [2–7]
Unprovoked PE, n (%)	10 (55)	52 (67.5)	19 (65.5)
**Laboratory investigations**			
Fibrinogen, g/L	3.08 [2.15–3.60]	3.33 [2.82–3.96]	3.22 [2.78–3.95]
CRP, mg/L	2.25 [1.48–6.24]*	3.62 [1.71–12.37]*	10.15 [2.63–23.34]*^#^
D-dimer, ng/mL	3,345 [2008–5983]	2,600 [1546–5053]	3,837 [2346–7770]
NT-proBNP, pg/mL	91.5 [59–104.5]*	444 [211–987]*^†^	1,261 [384–3571]*^‡#^
PAI-1, ng/mL	16.5 [11.4–23.3]*	22.7 [17.4–30]*	31 [19.8–45.8]*^#^
Citrullinated histone H3, ng/mL	2.28 [1.87–2.71]	2.92 [1.97–3.87]	3.44 [1.46–4.2]
ETP, nM × min	1508 [1436–1580]*	1683 [1499–1891]*^†^	1811 [1598–2395]*^#^
**Fibrin clot properties**			
K_s_, × 10^-9^cm^2^	7.3 [7.0–7.8]*	6.4 [5.6–7.2]*^†^	5.9 [4.8–7.0]*^#^
CLT, min	85 [79.5–90]*	108 [98–119]*^†^	122 [106–149]*^‡#^

*ACEI* angiotensin-converting enzyme inhibitors, *ARB* angiotensin II receptor blockers, *CLT* clot lysis time, *COPD* chronic obstructive pulmonary disease, *CRP* C-reactive protein, *ETP* endogenous thrombin potential, *K*_*s*_ fibrin clot permeability, *NT-proBNP* N-terminal B-type natriuretic propeptide, *PAI-1* plasminogen activator inhibitor type 1.

*p-value < 0.05 for low risk vs. intermediate-low risk vs. intermediate-high risk, respectively (post hoc: ^†^low risk vs. intermediate-low risk, ^‡^intermediate-low risk vs. intermediate-high risk, and ^#^low risk vs. intermediate-high risk).

sPESI score ≥ 2 was found in 65 (51.6%) patients, sPESI = 1 in 41 (2.5%), while 20 (15.9%) subjects had sPESI = 0 (Table S2). Levels of fibrinogen, CRP, NT-proBNP, and PAI-1 increased with sPESI score (Table 2). Interestingly, patients with sPESI score ≥ 2 compared to those with sPESI = 0 had 50% higher citH3 levels (Table S2). Higher ETP was observed in patients with sPESI ≥ 2 or 1 compared to low-risk patients (Table S2), with no intergroup differences in other haemostatic parameters. Subjects with sPESI score ≥ 2 or 1 had 35.3% and 16.5% longer CLT compared with low-risk patients with sPESI = 0, respectively (Table S2). Reduced K_s_ by 19.2% was observed in subjects with sPESI ≥ 2 compared to the former group (Table S2).

**Table 2:** Characteristics of acute PE patients with intermediate early mortality risk who died and survivors at 1-year follow-up.

Variable	PE-related death (n = 9)	Survivors (n = 97)	P value
Age, years	68 [61–80]	58 [40–70]	0.032
Men, n (%)	4 (44.4)	49 (50.5)	0.73
Body-mass index, kg/m^2^	26 [23–30]	27 [24–31]	0.28
**Characteristics of acute PE**			
Intermediate-low mortality risk, n (%)	2 (22.2)	75 (89.3)	0.0004
Intermediate-high mortality risk, n (%)	7 (77.8)	22 (26.2)	
Right ventricular dysfunction, n (%)	8 (88.9)	38 (39.2)	0.004
Unprovoked PE, n (%)	7 (77.8)	64 (66)	0.47
**Clinical characteristics, n (%)**			
Prior venous thromboembolism, n (%)	0	6 (6.2)	0.58
Coronary heart disease, n (%)	3 (33.3)	40 (41.2)	0.64
Prior myocardial infarction, n (%)	2 (22.2)	22 (22.7)	0.97
Prior stroke, n (%)	1 (11.1)	8 (8.2)	0.77
Hypertension, n (%)	3 (33.3)	56 (57.7)	0.16
Heart failure, n (%)	2 (22.2)	23 (23.7)	0.92
Diabetes mellitus, n (%)	3 (33.3)	33 (34)	0.97
Chronic obstructive pulmonary disease, n (%)	0	12 (12.4)	0.59
**Laboratory investigations**			
Endogenous thrombin potential, nM × min	2088 [1765–2593]	1688 [1499–1943]	0.031
Citrullinated histone H3, ng/mL	4.49 [3.66–5.62]	3.1 [1.9–3.98]	0.038
K_s_, × 10^-9^cm^2^	5.60 [4.90–5.91]	6.35 [5.45–7.2]	0.066
CLT, min	129 [115–149]	109 [99–124]	0.029

Data are shown as numbers (%) or median [1st quartile-3rd quartile].

*K*_*s*_ fibrin clot permeability, *CLT* clot lysis time.

### Associations of fibrin clot properties

**Figure 2:**
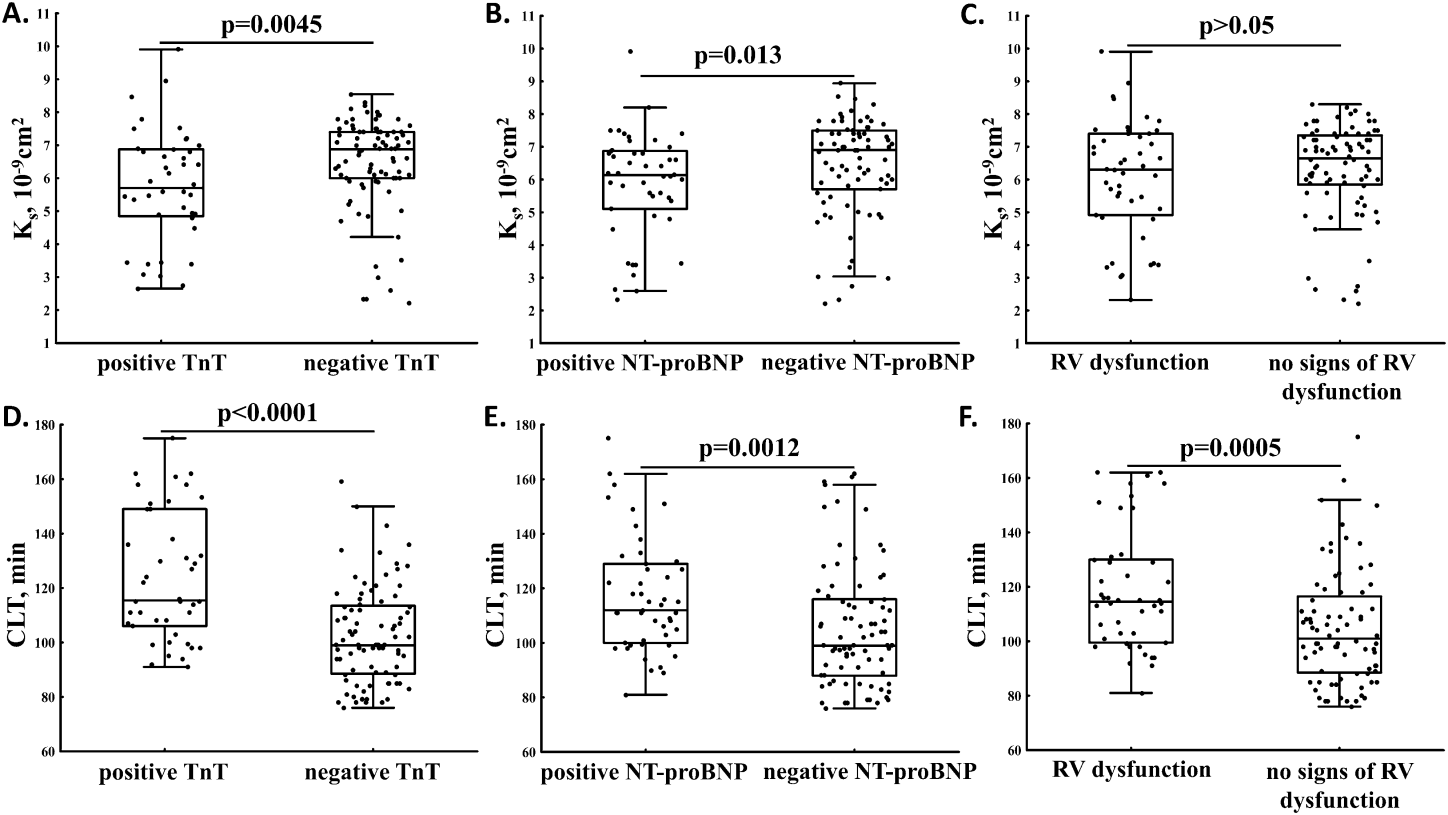
Fibrin clot permeability (K_s_) and clot lysis time (CLT) in acute PE patients with positive vs. negative TnT (**A**,**D**) and NT-proBNP (**B**,**E**) and with or without right ventricular (RV) dysfunction (**C**,**F**).

We found reduced K_s_ in patients with elevated TnT (Fig. 2A) and NT-proBNP (Fig. 2B), but not RV dysfunction (Fig. 2C). K_s_ was inversely associated with fibrinogen (r = − 0.32, p < 0.001), CRP (r = − 0.27, p = 0.0019), NT-proBNP (r = − 0.22, p = 0.012), and d-dimer (r = − 0.19, p = 0.038).

**Figure 3:**
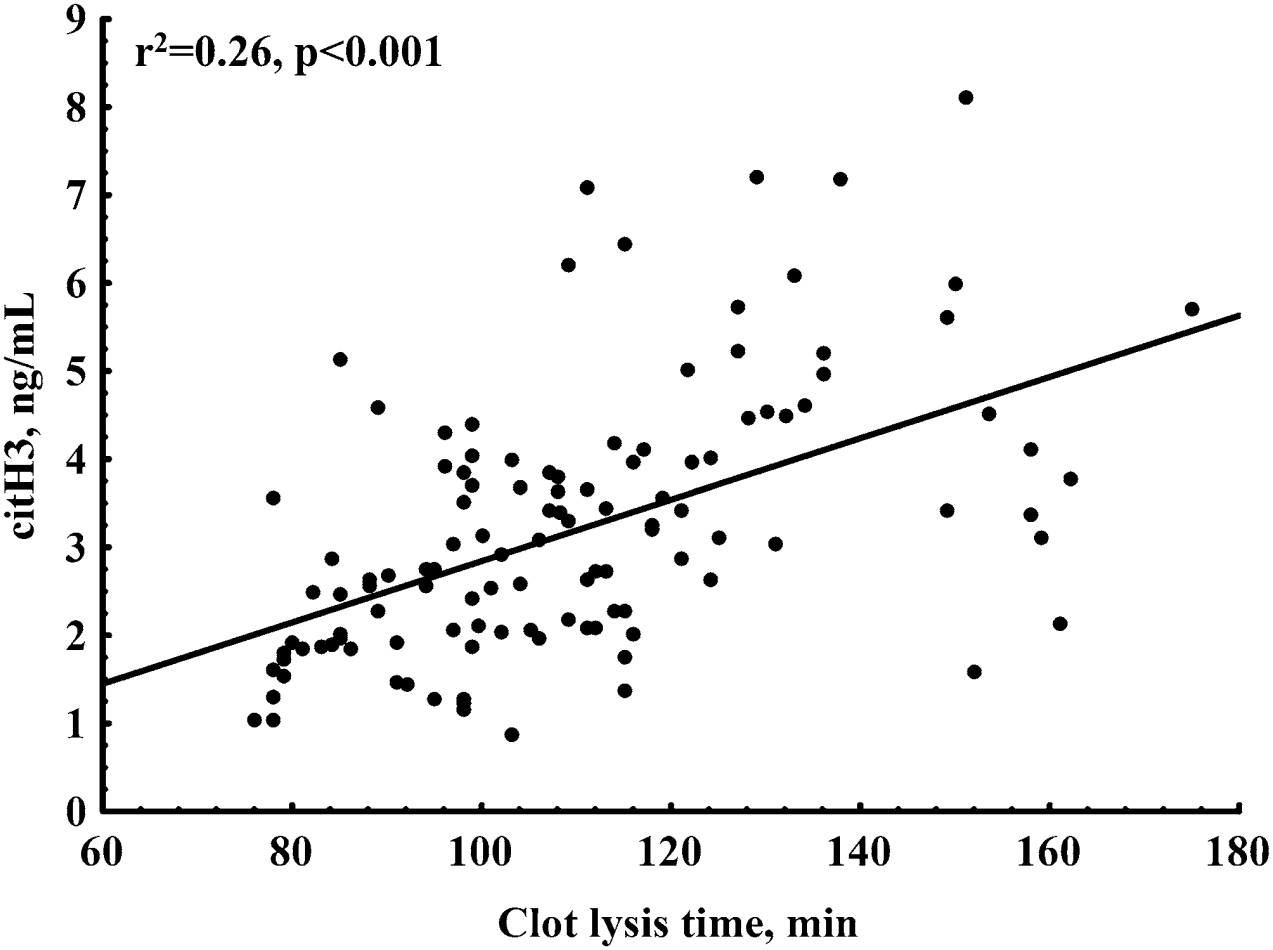
Association of clot lysis time with citrullinated histone H3 (citH3) levels in acute PE patients.

PE patients with positive TnT or NT-proBNP and those with RV dysfunction had prolonged CLT compared to the remainder (Fig. 2D–F). We observed linear associations of CLT with NT-proBNP (r = 0.29, p = 0.001) and ETP (r = 0.60, p < 0.001). Plasma citH3 concentrations correlated with CLT (Fig. 3) but not with K_s_.

### Predictors of clot density and lysability

After adjustment for age, sex, BMI, and fibrinogen levels, the independent predictors of CLT > 121.5 min (the top quartile, n = 32) were PAI-1 and citH3 levels (odds ratio [OR] = 1.09, 95% confidence interval [CI] 1.05–1.15 and OR = 1.80, 95% CI 1.26–2.70 per 1 unit increase, respectively), while K_s_ ≤ 5.46 × 10^–9^cm^2^ (the first quartile, n = 32) was predicted by CRP (OR = 1.49, 95% CI 1.16–2.10 per 10 units increase).

### Clot imaging

**Figure 4:**
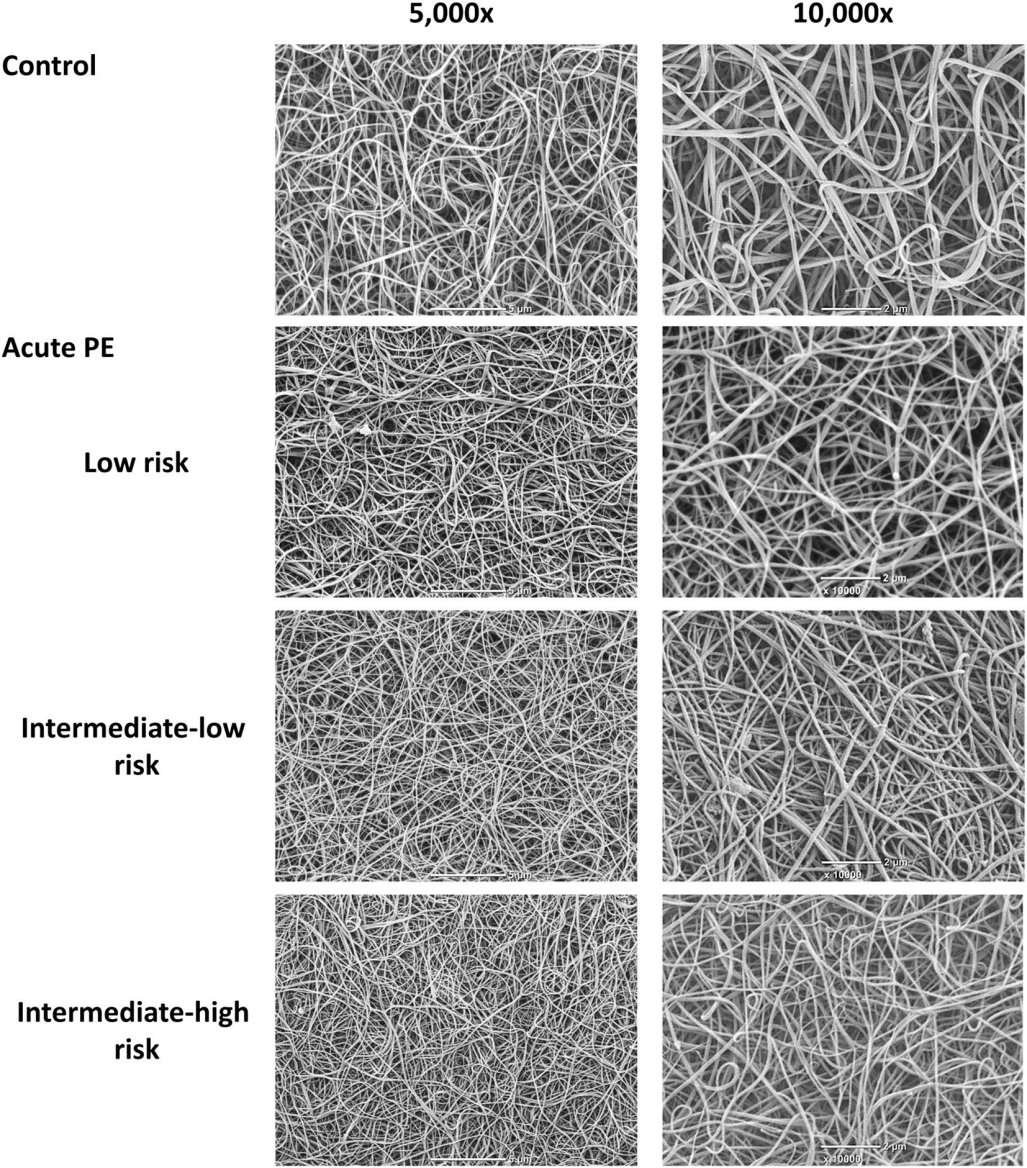
Representative scanning electron micrographs showing morphology of fibrin clots prepared from plasma of healthy control and acute PE patients with similar fibrinogen levels. Scale bar 5 or 2 µm.

SEM analysis performed in 28 clots prepared from plasma of healthy controls and patients with low, intermediate-low and intermediate-high early mortality risk (each, n = 7) revealed a smaller fibrin fibre diameter in PE patients compared to controls, especially in those with higher mortality risk (Fig. 4) in relation to lower K_s_. The corresponding median fibre diameter was 156 [149–174] nm, 141 [138–145] nm, 134 [130–136] nm, and 121 [119–126] nm, respectively (p < 0.01 for ANOVA).

### Mortality among PE patients

**Figure 5:**
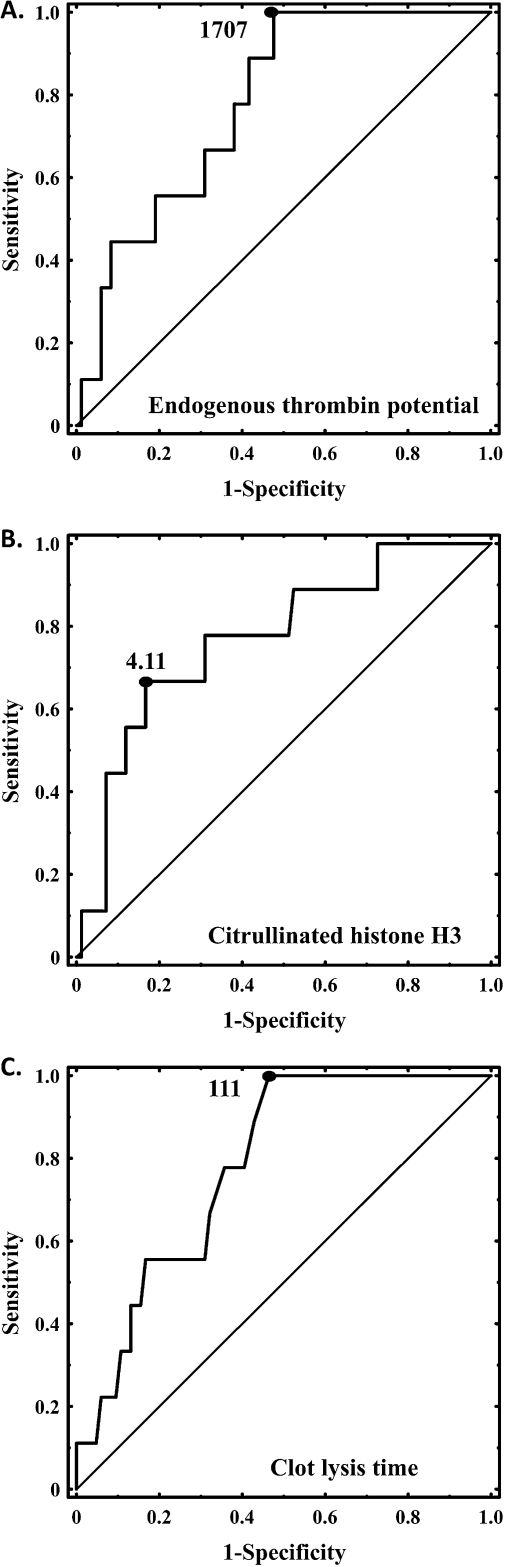
ROC curves for endogenous thrombin potential (**A**), citrullinated histone H3 (**B**), and clot lysis time (**C**). The outcome investigated in the ROC analysis was death during 1-year follow-up. Optimal cutoffs are presented.

All patients were on anticoagulation, mostly on rivaroxaban 20 mg/day. Two patients who died stopped anticoagulation with rivaroxaban due to trauma or invasive procedure prior to fatal PE. Five acute PE patients (3.97%) died within 30 days since admission, while four (3.2%) subjects died during a 1-year follow-up. Patients who died after acute PE event (n = 9, 7.14%) compared to survivors were older, more often had RV dysfunction, and were characterized by 24% higher ETP, 45% increased citH3 levels, and 18% prolonged CLT on admission (Table 2). A tendency to reduced K_s_ was also observed (p = 0.066; Table 2). Cut-off values which discriminated intermediate-risk PE patients who died from survivors were for ETP above 1707 ng/ml (sensitivity, 100% and specificity, 52%, area under curve [AUC] 0.779, 95% CI 0.653–0.905, p < 0.0001; Fig. 5A), for citH3 above 4.11 ng/ml (sensitivity, 67% and specificity, 83%, AUC 0.771, 95% CI 0.611–0.930, p = 0.0009; Fig. 5B), and for CLT above 111 min (sensitivity, 100% and specificity, 54%; AUC 0.782, 95% CI 0.662–0.901, p < 0.0001; Fig. 5C). Individuals with high ETP combined with elevated citH3 levels and prolonged CLT based on optimal cut-off values estimated using ROC analysis had the highest risk of PE-related death (OR, 9.3; 95% CI 2.1–40.3).

## Discussion

This study is the first to show that prothrombotic plasma fibrin clot properties accompanied by enhanced thrombin generation potential, increased PAI-1 concentrations, and interestingly enhanced NETs formation, characterize patients with more severe acute PE. Our novel finding is that prolonged CLT assessed in plasma on admission might help identify intermediate-risk patients with acute PE at increased risk of PE-related death. Efficiency of fibrinolysis using various plasma-based assays, including the most commonly used CLT, has been studied in patients with a history of acute thromboembolic event^8,10^. Its prognostic value has been reported in terms of recurrent thromboembolism^13,15,18,19^ and adverse outcomes following acute coronary syndrome^9^. The present study provides novel findings suggesting that measurement of CLT in acute PE might offer valuable information on short-term prognosis.

Denser fibrin networks observed in subjects with intermediate PE risk compared to those with low PE risk is a novel finding and deserves a comment. In our cohort of acute PE patients CRP predicted reduced K_s_. CRP has been reported to bind to fibrinogen and modify fibrin formation^8,20^. It might suggest that the architecture of plasma fibrin clots formed in vitro in acute PE is associated with the disease severity, indicating the mechanism which is driven by the inflammation, at least in part by NETs formation. Of note, recently it has been shown that fibrin structure may contribute to long-term complications after acute PE^21^. Both K_s_ and CLT were associated with positive TnT and NT-proBNP, biomarkers related to the cardiac injury, cardiac stress, and RV dysfunction in acute PE^3,22^. Levels of NT-proBNP have been shown to predict the 30-day adverse outcome in acute PE patients^23^. Of note, the 2019 ESC Guidelines for the diagnosis and management of acute PE downplayed the role of NT-proBNP in PE mortality risk estimation^3^, however, it is still considered as an additional prognostic biomarker. Our study showed that NT-proBNP could be a valuable marker of the prothrombotic and hypofibrinolytic state driven by increased thrombin generation and inflammation, similarly to patients with atrial fibrillation^24–26^. It is likely that the secondary prothrombotic effects associated with common consequences of acute clinical setting as enhanced inflammatory state or hemodynamic abnormalities are superimposed on the primary clot phenotype, which is determined by congenital and environmental factors present prior to acute PE^27^. This issue requires however further investigations.

We confirmed our hypothesis on the impact of NETs formation on acute PE, because in the present study, increased NETs formation, reflected by citH3 levels, was positively associated with impaired fibrinolysis. Our data suggests that NETs formation contributes to slower fibrinolysis a few days since the acute PE symptom onset. As shown by Longstaff et al.^28^, clots rich in DNA and histones are resistant to fibrinolysis. The relevance of NETosis in acute PE underscores our observation that elevated circulating citH3 in acute PE might be linked to disease severity by enhancing the inflammatory and prothrombotic state. Further large-scale studies on the role of NETs formation in acute VTE and its prognostic value should be conducted.

Our study has several limitations. First, the number of study participants was limited, however, the study was adequately powered to detect intergroup differences in clot properties. Second, high-risk PE patients and cancer patients were excluded, therefore our findings could not be extrapolated to these subsets. Cancer and pregnancy are potent prothrombotic factors negatively altering clot phenotype^29^. We decided to enrol women with contraceptives-related PE because it is not clear whether this type of PE differs from other types. Third, we evaluated fibrin clot properties using platelet-poor plasma, therefore the impact of platelets and blood cells cannot be assessed using this approach. Finally, due to limited number of patients the influence of different comorbidities on fibrin clot properties impairment in acute PE patients was difficult to assess.

In conclusion, prothrombotic fibrin clot properties in acute PE driven at least in part by enhanced NETs formation are associated with the PE severity. This suggests that a subgroup of normotensive noncancer PE patients with more compact clot structure and impaired fibrinolysis detected on admission should be under close clinical surveillance after discharge within the first year. Further studies on large cohorts of PE patients are needed to confirm our findings.

## Supplementary information

Supplementary information

## Data Availability

The datasets generated during and/or analysed during the current study are available from the corresponding author on reasonable request.

## References

[1] Silverstein MD (1998). Trends in the incidence of deep vein thrombosis and pulmonary embolism: a 25-year population-based study. Arch. Intern. Med..

[2] Konstantinides SV, Barco S, Lankeit M, Meyer G (2016). Management of pulmonary embolism: an update. J. Am. Coll. Cardiol..

[3] Konstantinides SV (2019). 2019 ESC Guidelines for the diagnosis and management of acute pulmonary embolism developed in collaboration with the European Respiratory Society (ERS): The Task Force for the diagnosis and management of acute pulmonary embolism of the European Society of Cardiology (ESC). Eur. Respir. J..

[4] Rali PM, Criner GJ (2018). Submassive pulmonary embolism. Am. J. Respir. Crit. Care Med..

[5] Kimball AS, Obi AT, Diaz JA, Henke PK (2016). The emerging role of NETs in venous thrombosis and immunothrombosis. Front. Immunol..

[6] Brill A (2012). Neutrophil extracellular traps promote deep vein thrombosis in mice. J. Thromb. Haemost..

[7] Savchenko AS (2014). Neutrophil extracellular traps form predominantly during the organizing stage of human venous thromboembolism development. J. Thromb. Haemost..

[8] Undas A, Ariëns RA (2011). Fibrin clot structure and function: a role in the pathophysiology of arterial and venous thromboembolic diseases. Arterioscler. Thromb. Vasc. Biol..

[9] Sumaya W (2018). Fibrin clot properties independently predict adverse clinical outcome following acute coronary syndrome: a PLATO substudy. Eur. Heart. J..

[10] Frączek P, Krzysztofik M, Stanisz A, Undas A (2019). Clinical outcomes and plasma clot permeability and lysability in patients with venous thromboembolism on rivaroxaban: a cohort study. Pol. Arch. Intern. Med..

[11] Undas A (2009). Altered fibrin clot structure/function in patients with idiopathic venous thromboembolism and in their relatives. Blood.

[12] Kupis RW, Goldman-Mazur S, Polak M, Ząbczyk M, Undas A (2019). Faster fibrin clot degradation characterizes patients with central pulmonary embolism at a low risk of recurrent peripheral embolism. Sci Rep..

[13] Zabczyk M, Plens K, Wojtowicz W, Undas A (2017). Prothrombotic fibrin clot phenotype is associated with recurrent pulmonary embolism after discontinuation of anticoagulant therapy. Arterioscler. Thromb. Vasc. Biol..

[14] Martinez MR (2014). Enhanced lysis and accelerated establishment of viscoelastic properties of fibrin clots are associated with pulmonary embolism. Am. J. Physiol. Lung Cell Mol. Physiol..

[15] Cieslik J, Mrozinska S, Broniatowska E, Undas A (2018). Altered plasma clot properties increase the risk of recurrent deep vein thrombosis: a cohort study. Blood.

[16] Jaff MR (2011). Management of massive and submassive pulmonary embolism, iliofemoral deep vein thrombosis, and chronic thromboembolic pulmonary hypertension: a scientific statement from the American Heart Association. Circulation.

[17] Pieters M (2018). An international study on the feasibility of a standardized combined plasma clot turbidity and lysis assay: communication from the SSC of the ISTH. J. Thromb. Haemost..

[18] Siudut J, Świat M, Undas A (2015). Altered fibrin clot properties in patients with cerebral venous sinus thrombosis: association with the risk of recurrence. Stroke.

[19] Meltzer ME (2010). Venous thrombosis risk associated with plasma hypofibrinolysis is explained by elevated plasma levels of TAFI and PAI-1. Blood.

[20] Ząbczyk M (2019). Plasma fibrin clot proteomics in healthy subjects: relation to clot permeability and lysis time. J. Proteom..

[21] Planquette B (2018). Fibrinogen and the prediction of residual obstruction manifested after pulmonary embolism treatment. Eur. Respir. J..

[22] Suzuki T (2016). Editor's Choice-Biomarkers of acute cardiovascular and pulmonary diseases. Eur. Heart J. Acute Cardiovasc. Care..

[23] Keller K (2019). Sex-specific differences in pulmonary embolism. Thromb Res..

[24] Matusik PT (2017). Elevated NT-proBNP is associated with unfavorably altered plasma fibrin clot properties in atrial fibrillation. Int. J. Cardiol..

[25] Matusik PT (2018). Association of cardiac troponin I with prothrombotic alterations in atrial fibrillation. Kardiol Pol..

[26] Wolberg AS (2007). Thrombin generation and fibrin clot structure. Blood Rev..

[27] Mirshahi S, Varin R, Soria J (2019). Importance of clot permeability and clot degradability for determination of rivaroxaban efficacy. Pol. Arch. Intern. Med..

[28] Longstaff C (2013). Mechanical stability and fibrinolytic resistance of clots containing fibrin, DNA, and histones. J. Biol. Chem..

[29] Ząbczyk M (2019). Altered fibrin clot properties in advanced lung cancer: strong impact of cigarette smoking. Med. Oncol..

