# Supplementary material for: Prothrombotic fibrin clot properties associated with NETs formation characterize acute pulmonary embolism patients with higher mortality risk
Source: Sci Rep. 2020 Jul 10;10:11433. doi: 10.1038/s41598-020-68375-7 (PMC7351737; doi:10.1038/s41598-020-68375-7)
Supplement: Supplementary file 1 — Supplementary information [file 41598_2020_68375_MOESM1_ESM.docx]

**Supplemental Data**

**Prothrombotic fibrin clot properties associated with NETs formation characterize acute pulmonary embolism patients with higher mortality risk**

Michał Ząbczyk, PhD^a,b^, Joanna Natorska, PhD^a,b^, Agnieszka Janion-Sadowska, MD, PhD^c^, Agnieszka Metzgier-Gumiela, MD, PhD^d^, Mateusz Polak, MD, PhD^e^, Krzysztof Plens, MSc^f^, Marianna Janion, MD, PhD^c^, Grzegorz Skonieczny, MD, PhD^d^, Katarzyna Mizia-Stec, MD, PhD^e^, Anetta Undas, MD, PhD^a,b,c^

a. Institute of Cardiology, Jagiellonian University Medical College, Krakow, Poland, b. John Paul II Hospital, Krakow, Poland, c. The Faculty of Medicine and Health Sciences, The Jan Kochanowski University, Kielce, Poland d. Provincial Polyclinical Hospital, Torun, Poland, e. First Department of Cardiology, Leszek Giec Upper-Silesian Medical Centre of the Silesian Medical University in Katowice, Poland, and f. KCRI, Krakow, Poland

**Supplemental Methods**

***Patients***

RV dysfunction was defined as dilatation of the RV (a RV/left ventricle diameter ratio of

>1.0 from the sub-costal or apical view four-chamber view) combined with the absence of the

inspiratory collapse of the inferior vena cava or an elevated systolic gradient through the tricuspid valve (>30 mm Hg), in the absence of significant left ventricular disease.

The diagnosis of DVT was established by a positive finding of color duplex sonography (visualization of an intraluminal thrombus in calf, popliteal, femoral or iliac veins) performed within the first 48 hours since enrolment. Unprovoked VTE episode was defined as having no history of cancer, surgery requiring general anesthesia, major trauma, plaster cast or hospitalization in the past month, pregnancy or delivery in the past 3 months. Heart failure (HF) was defined as the presence of relevant symptoms and signs and left ventricular ejection fraction ≤45%. Chronic obstructive pulmonary disease (COPD) was defined as a disorder characterized by abnormal tests of expiratory flow.

***Laboratory investigations***

Immunoenzymatic assays were used to determine in citrated plasma plasminogen activator inhibitor-1 (PAI-1) antigen and citrullinated histone H3 (citH3) (Cayman Chemical, Ann Arbor, MI, USA).

***Fibrin permeation analysis***
Fibrin clot permeation was determined using a pressure-driven system. Briefly, 20 mM calcium chloride and 1 U/mL human thrombin (Merck KGaA, Darmstadt, Germany) were added to 120 µl citrated plasma. After 2 hours of incubation in a wet chamber, tubes containing the clots were connected via plastic tubing to a reservoir of a buffer (0.01 M Tris, 0.1 M NaCl, pH 7.5) and its volume flowing through the gels was measured within 60 minutes. A permeation coefficient (K_s_), which indicates the pore size, was calculated from the equation: K_s_=QxLxη/txAxΔp, where Q is the flow rate in time t; L, the length of a fibrin gel; η, the viscosity of liquid (in poise); A, the cross-sectional area (in cm^2^) and Δp, a differential pressure (in dyne/cm^2^).

***Scanning Electron Microscopy (SEM)***

After K_s_ measurement, clots (n=21) were fixed using 2.5% glutaraldehyde, washed with distilled water, dehydrated in graded water-ethanol solutions, dried by the critical point procedure and sputter coated with gold. Samples were scanned in 10 different areas (microscope JEOL JCM-6000; JEOL Ltd., Tokyo, Japan) at magnification of 5,000x and 10,000x to determine a fibrin diameter of at least 50 fibers per clot using the ImageJ software (US National Institutes of Health, Bethesda, MD, USA).

***Plasma clot lysis assay***

To assess efficiency of clot lysis, clot lysis time (CLT) was used. Briefly, citrated plasma was mixed with 15 mM calcium chloride, 0.5 U/mL human thrombin (Merck), 15 µM phospholipid vesicles (Rossix, Mölndal, Sweden) and 20 ng/mL recombinant tPA (rtPA, Boehringer Ingelheim, Germany). The mixture was transferred to a microtiter plate and its turbidity was measured at 405 nm at 37°C. CLT was defined as the time from the midpoint of the clear-to-maximum-turbid transition, which represents clot formation, to the midpoint of the maximum-turbid-to-clear transition (representing the lysis of the clot).

***Endogenous thrombin potential***

Endogenous thrombin potential (ETP) was performed using calibrated automated thrombography (Thrombinoscope BV, Maastricht, the Netherlands) according to the manufacturer’s instructions in the 96-well plate fluorometer (Ascent Reader, Thermolabsystems OY, Helsinki, Finland), equipped with the 390/460 filter set, at a temperature of 37^o^C. Briefly, 80 µL of platelet-poor plasma were diluted with 20 µL of the reagent containing 5 pM recombinant TF, 4 micro-molar phosphatidylserine/phosphatidylcholine/phosphatidylethanolamine vesicle, and 20 µL of FluCa solution (HEPES, pH 7.35, 100 nM CaCl_2_, 60 mg/mL bovine albumin, and 2.5 mM Z-Gly-Gly-Arg-7-amino-4-methylcoumarin). Each plasma sample was analyzed in duplicate. For analysis, the area under the curve, expressed as ETP, was used.

***Statistical analysis***

Univariate and multivariate logistic regression models were performed to identify independent predictors of prolonged CLT or reduced K_s_. The multivariate model was fitted using backward stepwise regression. Variables that were associated with the prolonged CLT or reduced K_s_ with a significance level of p<0.2 in the bivariate models were selected for possible inclusion in the multivariate logistic regression models. Multivariate models were adjusted for age, sex, body-mass index (BMI), and fibrinogen levels. The best cut-off value that maximizes sensitivity and specificity and differentiates PE patients who died from survivors was calculated by using the Receiver Operating Characteristics (ROC) curve. The study was powered to have a 90% chance of detecting a 10% difference in fibrin clot characteristics using a p-value of 0.05. In order to demonstrate such a difference or greater, 19 patients were required in the group. In turn, to demonstrate such a difference in K_s_ using a p-value of 0.05, at least 26 patients were required in the group.

**Table S1.** Baseline characteristics of acute pulmonary embolism patients compared to controls.

| **Variable** | **Healthy controls,**  **n=25** | **Acute PE patients,  n=126** |  | **P-value** |
| --- | --- | --- | --- | --- |
| Age, years | 55.9±27.4 | 58.2±14.4 |  | 0.30 |
| Sex (male), n (%) | 13 (52) | 66 (52.4) |  | 0.99 |
| Body-mass index, kg/m^2^ | 24.8±3.0 | 28.1±5.1 |  | 0.0014 |
| **Laboratory investigations** | |  |  |  |
| White blood cell count, 10^3^/µL | 6.12 [5.27-7.97] | 7.03 [5.50-9.18] |  | 0.13 |
| Neutrophil count, 10^3^/µL | 3.44 [2.75-4.30] | 3.82 [3.10-5.77] |  | 0.07 |
| Platelet count, 10^3^/µL | 254 [235-315] | 220 [191-283] |  | 0.01 |
| Fibrinogen, g/L | 3.03 [2.91-3.44] | 3.26 [2.76-3.88] |  | 0.47 |
| hsCRP, mg/L | 1.67 [0.74-2.61] | 3.65 [1.70-12.50] |  | <0.001 |
| D-dimer, ng/mL | 309 [269-441] | 3233 [1661-5325] |  | <0.001 |
| NT-proBNP, pg/mL | 94 [75-107] | 399 [106-1045] |  | <0.001 |
| PAI-1, ng/mL | 15.0 [7.9-19.5] | 22.9 [16.7-33.2] |  | <0.001 |
| Citrullinated histone H3, ng/mL | 0.59 [0.14-0.78] | 2.77 [1.90-3.98] |  | <0.001 |
| ETP, nM×min | 1176 [864-1274] | 1660 [1494-1894] |  | <0.001 |
| K_s_, ×10^-9^cm^2^ | 7.78 [6.99-8.11] | 6.50 [5.46-7.40] |  | <0.001 |
| CLT, min | 84.8 [75.0-98.5] | 106.5 [95.0-121.6] |  | <0.001 |

Data are shown as numbers (%), mean±standard deviation or median [1st quartile-3rd quartile]. Abbreviations: hsCRP, High-sensitivity C-reactive protein; NT-proBNP, N-terminal B-type natriuretic propeptide, PAI-1, plasminogen activator inhibitor; ETP, endogenous thrombin potential; K_s_, fibrin clot permeability, CLT, clot lysis time.

**Table S2.** Characteristics of patients with acute pulmonary embolism (PE) according simplified PE severity index (sPESI).

| **Variable** | **sPESI score** | | |
| --- | --- | --- | --- |
|  | **0**  **(n=20)** | **1**  **(n=41)** | **≥2**  **(n=65)** |
| Age, years | 55±12.7 | 57±12.2 | 58.2±14.4 |
| Men, n (%) | 13 (65) | 21 (51.2) | 32 (49.2) |
| Body-mass index, kg/m^2^ | 28.6±5.2 | 28.3±5.3 | 28.0±4.9 |
| Current smoking, n (%) | 4 (20) | 8 (19.5) | 13 (20) |
| **Clinical characteristics, n (%)** | | | |
| Prior venous thromboembolism | 3 (15) | 2 (4.9) | 4 (6.2) |
| Coronary heart disease | 7 (35) | 16 (39) | 27 (41.5) |
| Prior myocardial infarction | 1 (5) | 12 (29.3) | 12 (18.5) |
| Prior stroke | 1 (5) | 3 (7.3) | 6 (9.2) |
| Hypertension | 9 (45) | 21 (51.2) | 38 (58.4) |
| Heart failure | 0* | 12 (29.3)*† | 13 (20)*# |
| Diabetes mellitus | 6 (30) | 13 (31.7) | 23 (35.4) |
| COPD | 0 | 5 (12.2) | 7 (10.8) |
| **Medications, n (%)** | | | |
| Beta blockers | 20 (100)* | 27 (65.9)*† | 44 (67.7)*# |
| ACEI | 12 (60) | 24 (58.5) | 38 (58.5) |
| ARB | 5 (25) | 7 (17.1) | 4 (6.2) |
| Calcium channel blockers | 1 (5) | 5 (12.2) | 10 (15.4) |
| Aspirin | 5 (25) | 15 (36.6) | 20 (30.8) |
| Statin | 13 (65) | 27 (65.9) | 37 (56.9) |
| **Characteristics of acute PE** | | | |
| PE symptoms, days | 3.5 [1.5-7] | 3 [2-7] | 4 [2-7] |
| Unprovoked PE, n (%) | 10 (55) | 27 (65.9) | 44 (67.7) |
| **Laboratory investigations** | | | |
| Fibrinogen, g/L | 3.08 [2.15-3.60]* | 3.11 [2.65-3.69]* | 3.40 [2.96-4.22]*‡# |
| CRP, mg/L | 2.25 [1.48-6.24]* | 2.76 [1.71-6.12]* | 7.12 [2.14-23.86]*‡# |
| D-dimer, ng/mL | 3345 [2008-5983]* | 2123 [1440-4142]* | 3628 [1962-6486]*‡ |
| NT-proBNP, pg/mL | 91.5 [59-104.5]* | 287 [154-480]*† | 987 [345-1824]*‡# |
| PAI-1, ng/mL | 16.5 [11.4-23.3]* | 22.1 [16.7-30.0]*† | 27.5 [18.4-38.1]*‡# |
| Citrullinated histone H3, ng/mL | 2.28 [1.87-2.71]* | 2.70 [1.88-3.85]* | 3.42 [2.10-4.31]*# |
| ETP, nM×min | 1508 [1436-1580]* | 1599 [1437-1759]* | 1788 [1633-2097]*‡# |
| **Fibrin clot properties** | | | |
| K_s_, ×10^-9^cm^2^ | 7.3 [7.0-7.8]* | 7.0 [6.1-7.5]* | 5.9 [4.5-4.8]*‡# |
| CLT, min | 85 [79.5-90]* | 99 [94-109]*† | 115 [108-131]*‡# |

Data are shown as numbers (%), mean±standard deviation or median [1^st^ quartile-3^rd^ quartile].

*p-value <0.05 for sPESI=0 vs. sPESI=1 vs. sPESI≥2, respectively (post hoc: †sPESI=0 vs. sPESI=1, ‡sPESI=1 vs. sPESI≥2, and #sPESI=0 vs. sPESI≥2)

Abbreviations: ACEI, angiotensin-converting enzyme inhibitors; ARB, angiotensin II receptor blockers; CLT, clot lysis time; COPD, chronic obstructive pulmonary disease; CRP, C-reactive protein; ETP, endogenous thrombin potential; K_s_, fibrin clot permeability; NT-proBNP, N-terminal B-type natriuretic propeptide; PAI-1, plasminogen activator inhibitor type 1
